# Over time evaluation of glycaemic control in direct‐acting antiviral‐treated hepatitis C virus/diabetic individuals with chronic hepatitis or with cirrhosis

**DOI:** 10.1111/liv.14905

**Published:** 2021-05-26

**Authors:** Irene Cacciola, Giuseppina Russo, Roberto Filomia, Concetta Pitrone, Gaia Caccamo, Annalisa Giandalia, Angela Alibrandi, Maria Stella Franzè, Serena Porcari, Sergio Maimone, Carlo Saitta, Giovanni Squadrito, Giovanni Raimondo

**Affiliations:** ^1^ Department of Clinical and Experimental Medicine University of Messina Messina Italy; ^2^ Division of Medicine and Hepatology Department of Internal Medicine University Hospital of Messina Messina Italy; ^3^ Division of Medicine and Diabetology Department of Internal Medicine University Hospital of Messina Messina Italy; ^4^ Department of Economics Unit of Statistical and Mathematical Science of Messina University of Messina Messina Italy; ^5^ Division of Internal Medicine Department of Internal Medicine University Hospital of Messina Messina Italy

**Keywords:** chronic hepatitis C, DAA therapies, glycated haemoglobin, HCV‐related cirrhosis, type 2 diabetes mellitus

## Abstract

**Background:**

Data concerning the impact of hepatitis C virus (HCV) cure on type 2 diabetes mellitus (T2DM) are controversial. The aim of the study was to evaluate the effects of anti‐HCV direct‐acting antiviral (DAA) treatments on long‐term glucose control in HCV/T2DM patients with chronic hepatitis C (CHC) or with cirrhosis.

**Methods:**

One hundred and eighty‐two consecutive HCV/T2DM patients who achieved a viral clearance by DAA treatment were enrolled. Seventy or 182 of them had CHC, and 112 had cirrhosis. Clinical, biochemical and instrumental parameters were recorded at baseline and at 48, 96 and 120 weeks (48w, 96w and 120w, respectively) after stopping DAA therapy.

**Results:**

At baseline, the overall study population had a mean of glycated haemoglobin (HbA1c) value of 7.2% (ranging from 5 to 11.2), without any significant differences between CHC and cirrhosis [7.1 and 7.2, respectively]. Evaluation over time of HbA1c variations showed a significant improvement of glucose control at all post‐treatment time points compared with baseline in CHC patients (*P* = .001). In cirrhotic patients, a significant decrease of HbA1c levels was only found when comparing HbA1c values between baseline and 48w time‐point (*P* = .001), whereas this improvement disappeared at both 98w and 120w (*P* = .8 and *P* = .3, respectively). Multivariate logistic regression analysis showed that patients with chronic hepatitis have a 2.5 (CI 1.066‐5.945) times greater chance of achieving an improvement of glycaemic values than patients with liver cirrhosis (*P* = .035).

**Conclusion:**

DAA‐based HCV cure induces a significant and persistent amelioration of glycaemic control in HCV/diabetic patients with chronic hepatitis, whereas cirrhotic HCV/diabetic subjects have only a transient benefit from the virus elimination.

**Abbreviations**
ALT: alanine aminotransferase
AST: aspartate aminotransferase
AU: alcoholic unit
CHC: chronic hepatitis C
C‐P: Child–Pugh
CV: cardiovascular
DAA: direct‐acting antiviral
EGS: endoscopy
FBG: fasting blood glucose
GGT: gamma glutamyl transpeptidase
Hb: haemoglobin
HbA1c: glycated haemoglobin
HCC: hepatocellular carcinoma
HCV: hepatitis C virus
PLT: platelets
RBC: red blood cells
T2DM: type 2 diabetes mellitus
WBC: white blood cells

**Key points**

- Chronic hepatitis C patients with type 2 diabetes show persistent amelioration of glycaemic control after DAA‐based HCV cure
- Glycaemic control is only transiently improved after DAA‐based cure in HCV cirrhotic patients with type 2 diabetes

## 1 INTRODUCTION

Hepatitis C virus (HCV) is a major cause of liver diseases worldwide.^1^ Furthermore, HCV has also been associated with a relatively large spectrum of extra‐hepatic clinical disorders, including type 2 diabetes mellitus (T2DM).^2^, ^3^ In fact, epidemiological data show that about one third of patients with chronic HCV infection have T2DM.^4^, ^5^ Coexistence of chronic hepatitis C (CHC) and T2DM is associated with an increased risk of occurrence of the most severe long‐term sequelae of liver including cirrhosis and hepatocellular carcinoma (HCC).^6^, ^7^ In addition, the risk of cardiovascular (CVD) events in T2DM patients is increased in HCV‐infected patients.^8^ The recent availability of direct acting antivirals (DAAs) capable of inducing the permanent clearance of HCV in virtually all treated patients with no or minimal side effects has dramatically changed the evolution of HCV‐related liver disease, up to a complete clinical recovery in at least those cases in which cirrhosis has not yet developed. At present, however, data concerning the possible impact of DAAs on the HCV‐related extra‐hepatic manifestations are still insufficient.^9^, ^10^, ^11^ In particular, available studies concerning the effects of DAA treatments on glucose control in T2DM subjects are not only numerically limited but also divergent from each other.^12^, ^13^, ^14^ Indeed, the studies performed so far in this field have demonstrated several limitations including short‐term follow‐up and lack of information on possible differences related to the degree of the liver damage in the DAA‐treated patients.^15^, ^16^, ^17^ The aims of this study were to evaluate whether the DAA‐based HCV cures may have an effect on long‐term glucose control in T2DM patients and whether this possible effect might be influenced by the grade of severity of the liver disease.

## 2 PATIENTS AND METHODS

The design of this prospective study was to evaluate over time the glycaemic control in HCV/T2DM patients with severe but still compensated liver disease after successful DAA cure. Prescription of interferon‐free, DAA‐based anti‐HCV therapies has been available in Italy since April 2015, in accordance with the treatment initially limited to some categories of patients. In particular, subjects with cirrhosis in class A of Child–Pugh (C‐P) score or with advanced grade of chronic hepatitis (viz., F3 CHC) represented the vast majority of patients that could be treated.^18^ In accordance with the aim of the study, we enrolled all HCV/T2DM patients under anti‐diabetic therapy and with F3 CHC or with C‐P class‐A cirrhosis consecutively attending the Liver and the Diabetes Units of University Hospital of Messina and who underwent DAA treatments from 1 April 2015 to 31 December 2016. Exclusion criteria were hepatitis B virus or immunodeficiency virus coinfection, and—by definition—signs of liver decompensation (ie, ascites, porto‐systemic encephalopathy and hyperbilirubinemia). At baseline, diagnosis and staging of the liver disease were performed according to the established clinical, ultrasonographic, elastographic, endoscopic and biochemical criteria^19^ and confirmed by histology in the cases that had undergone liver biopsy in that period of time. In accordance with Agenzia Italia del FArmaco prescription limitations, all F3 CHC patients had liver stiffness values between 10 and 12 kPa, and all cirrhotic patients had liver stiffness values higher than 12 kPa at FibroScan evaluation. Additional criteria for making diagnosis of cirrhosis were a previous liver biopsy showing stage 4 fibrosis, presence of oesophageal and/or gastric varices at endoscopy (EGS) examination, platelet count lower than 100 × 10^3^/mmc, and typical ultrasound features.^20^, ^21^, ^22^ According to the design of the study, the following data were collected from each patient and recorded in a data set at baseline: demographic data [age, sex and body mass index (BMI)], presence of comorbidities (arterial hypertension, CVD and kidney diseases), virological data (serum HCV RNA amount and virus genotype), diabetes family history, diabetes duration, hypoglycaemic treatments, number of diabetes medications, diabetes complications, liver stiffness values measured by FibroScan, and haematological and biochemical data [counts of red blood cells (RBC), white blood cells (WBC), and platelets (PLT); values of haemoglobin (Hb), serum alanine aminotransferase (ALT), aspartate aminotransferase (AST), gamma glutamyl transpeptidase (GGT), bilirubin, cholesterol, triglycerides, albumin and gamma‐globulin, creatinine, international normalized ratio (INR), fasting plasma glucose (FBG), and glycated haemoglobin (HbA1c)]. Furthermore, alcohol intake was evaluated and quantified as the number of units of drinks per day [one drink or alcoholic unit (AU) = 12.5 g of pure ethanol contained in a glass of wine, a pint of beer, or a mini‐glass of spirits].^23^ According to the amount of alcohol intake, patients were divided in ‘teetotal or no consumption’ within at least 3 years before starting the study, ‘mild drinkers’ (up to 2 AU), and ‘heavy drinkers’ (more than 2 AU). All enrolled patients who obtained sustained virological response (SVR) (defined by a negative HCV‐RNA measurement 12 weeks after the end of DAA treatment) were systematically followed up at the outpatients clinics of both the liver and diabetes units. All the above listed clinical, ultrasonographic, elastographic, endoscopic and biochemical data as well as possible changes of anti‐diabetic treatment were also recorded at Weeks 48, 96 and 120 (48w, 96w and 120w, respectively) after stopping DAA therapies. The study was approved by the ethical committee of the district of Messina, and all patients signed informed consent.

## 3 STATISTICAL ANALYSIS

The numerical data were expressed as median and range (minimum and maximum) and the categorical variables as number and percentage.

The non‐parametric approach was used because most of the numerical variables were not normally distributed, such as verified by Kolmogorov–Smirnov test. The existence of significant differences between CHC and cirrhotic patients was evaluated using Mann–Whitney test (for numerical parameters) and chi‐square test or exact Fisher test or likelihood ratio test, as appropriate (for categorical variables). For each group, comparison of the clinical characteristics between baseline and the end of the study was performed using Wilcoxon test (for numerical parameters) and Mc Nemar test (for dichotomous variables). In particular, we used Wilcoxon rank sum test when in addition to considering whether each observation is below or above a value of interest, we want to take into account the dimension of the observations. The same tests were used to perform a paired comparison of clinical characteristics between baseline versus 48, 96 and 120 weeks, respectively.

Finally, univariate and multivariate logistic regression models were estimated in order to identify significant predictive factors of glucose control. The covariates were gender, age, BMI, stage of liver fibrosis (chronic hepatitis vs cirrhosis), HCV genotypes, presence of varices, albumin, gamma globulin, creatinine, AST ALT, GGT, liver decompensation, diabetes duration, diabetes family history, diabetes complications, and insulin treatment. The results were expressed as odds ratio (OR) with relative 95% confidence interval (CI) and *P* value.

Statistical analyses were performed using SPSS 22.0 for Windows package.

A *P* value lower than .05 was considered to be statistically significant.

## 4 RESULTS

**FIGURE 1:**
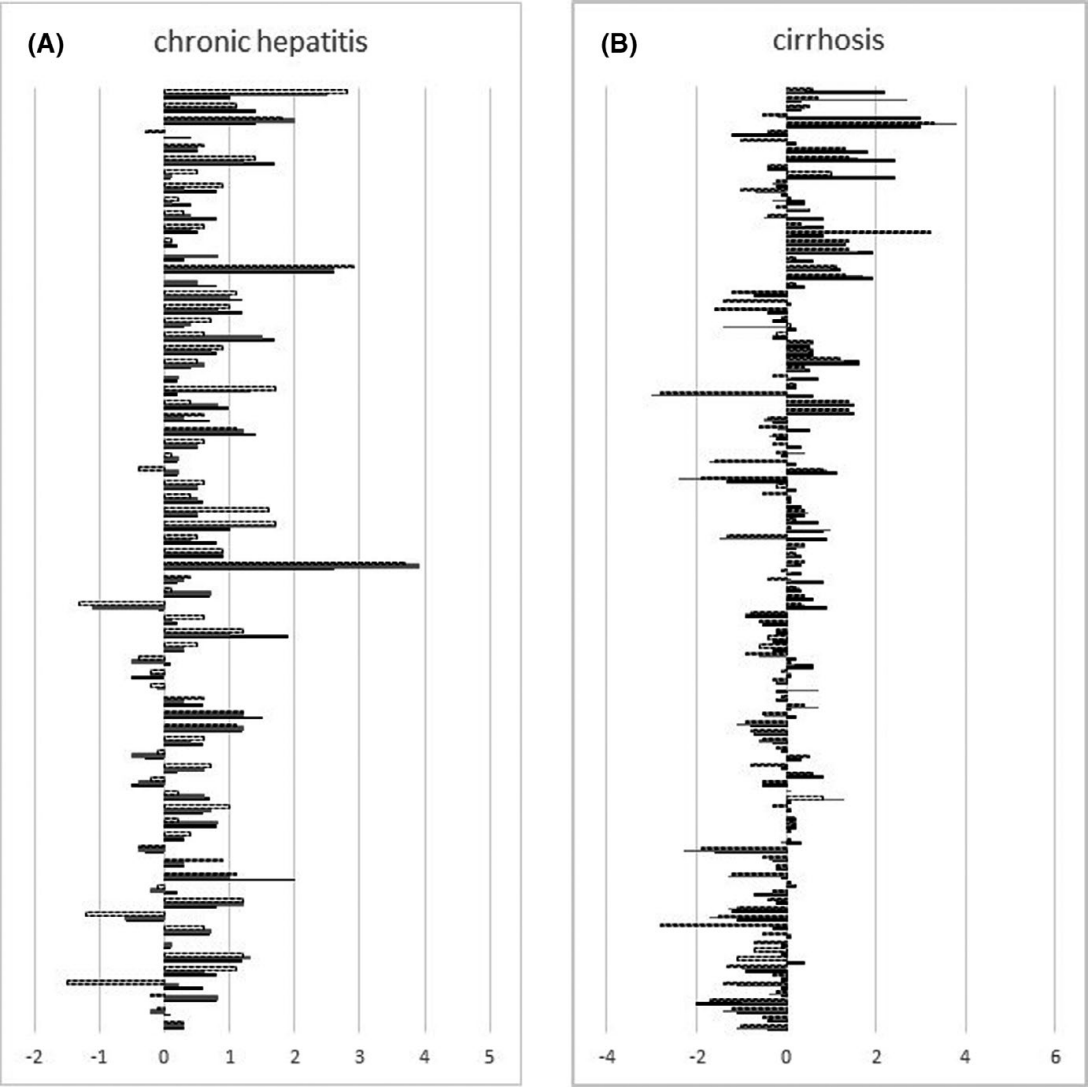
Haemoglobin (HbA1c) level variations in respect to the baseline values at 48 (bold line), 96 (grey line) and 120 (dotted line) weeks after stopping direct‐acting antiviral (DAA) therapies in chronic hepatitis (A) and cirrhotic (B) patients, (*P* < .001 by Wilcoxon analysis)

**FIGURE 2:**
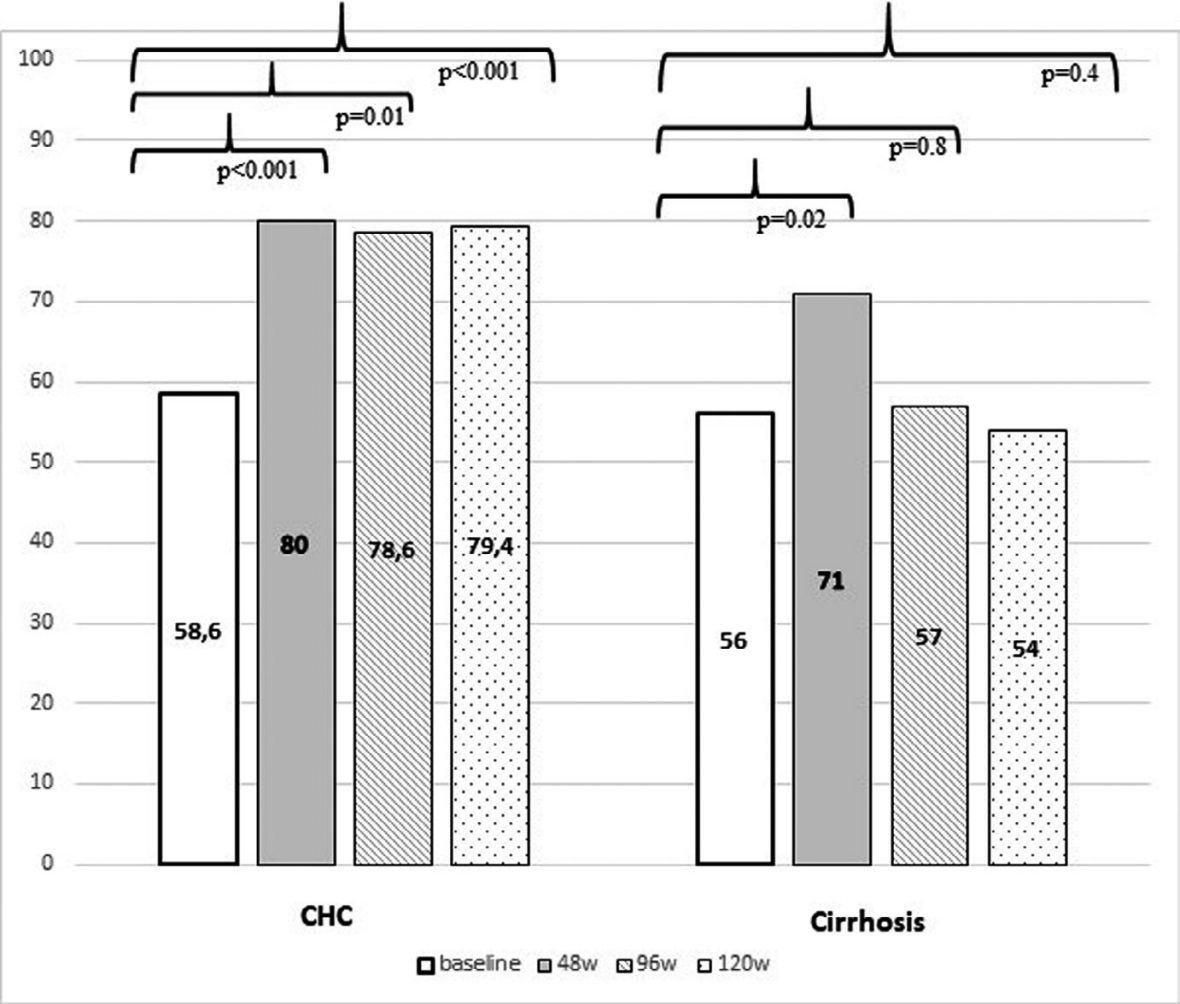
Percentage of 70 patients with chronic hepatitis C (CHC) and 112 with cirrhosis showing haemoglobin (HbA1c) values < 7.2 g/dL at baseline, and at 48, 96 and 120 weeks after stopping direct‐acting antiviral (DAA) treatments

Six hundred and forty‐seven consecutive HCV patients [383 (59.2%) males; median age 68.0, range 25‐93] with either F3 CHC (264 cases) or with C‐P class A cirrhosis (383 cases) underwent DAA treatment at the Division of Medicine and Hepatology of the University Hospital of Messina from 1 April 2015 and 31 December 2016. One hundred and eighty‐six (28.7%) of them had T2DM, also attended the Diabetic Unit of the University Hospital of Messina and were under anti‐diabetic therapy at the time of DAA treatment starting. Eighteen (9.9%) out of the 186 patients were infected with HCV genotype 1a, 107 (58.8%) with genotype 1b, 38 (20.9%) with genotype 2, 4 (2.2%) with genotype 3 and 11 (6%) with genotype 4. The patients were treated with the following DAA regimens: 71 Sofosbuvir (SOF) and Daclatasvir combination [plus ribavirin (RBV) in six cases]; 83 SOF and Lepidasvir combination (plus RBV in four cases); 12 Ombitasvir/Paritaprevir/ritonavir plus Dasabuvir; 16 SOF and Simeprevir combination plus RBV. One hundred and eighty‐two out of the 186 (98%) patients had SVR to DAA therapies and were included in the study. At baseline, 107 of 182 (58.8%) patients had arterial hypertension, and 12 (6.6%) had a history of CVD (eight had coronary heart disease; four cerebrovascular disease as defined by the European Society of Cardiology).^24^ Concerning the T2DM treatments, 105 (57.7%) subjects were on oral therapies, and 77 (42.3%) on insulin treatment, either alone (71 cases) or in combination (6 cases) with oral hypoglycaemic drugs. According to the amount of alcohol intake, 95 (52.2%) patients were teetotal or had no consumption within at least 3 years before starting DAA treatments; 63 (34.6%) were mild drinkers (up to 2 AU); 24 (13.2%) were heavy drinkers (more than 2 AU). Seventy out of 182 patients [38.4%; 35 males, median age 67 years (range 44‐83), median BMI 25.8 kg/m^2^ (range 18.7‐37.8)] had CHC with median liver stiffness values 11.1 kPa (range 10‐12 kPa). One hundred and twelve (61.4%) patients had C‐P class A cirrhosis [64 males, median age 68 years (range 44‐83); median BMI 26.6 kg/m^2^ (range 19.6‐37.1), median liver stiffness values 23.7 kPa (range 12.8‐65 kPa) (Table 1). At baseline, the overall study population had a mean HbA1c value of 7.2% (ranging from 5 to 11.2), without any significant differences between CHC and cirrhosis subgroups [7.1 (range 5.2‐10.8) and 7.2 (range 5.0‐11.2) (*P* = .8), respectively]. Similarly, no statistically significant differences were observed between the two subgroups concerning age, sex, BMI, infecting HCV genotype, presence of arterial hypertension, alcohol intake, T2DM therapeutic treatments, diabetes family history, diabetes duration, fasting blood glucose (FBG) values, number of diabetes medications, diabetes complications, serum levels of creatinine, ALT, AST, GGT, bilirubin and cholesterol values (Table 1). Over time assessment of glucose control evaluation of HbA1c value variations showed a significant improvement of glucose control compared with baseline at time points 48w, 96w and 120w (*P* < .001), despite there being no substantial changes in anti‐diabetic drugs used. However, when the analysis was subgrouped according to the degree of liver disease, CHC subgroup showed a persistent amelioration of HbA1c values in all three post‐treatment time points compared with baseline (*P* < .001), whereas cirrhosis subgroup showed a significant decrease of HbA1c values only at the 48w time point (*P* = .001), but such an improvement disappeared at both 98w and 120w (*P* = .8 and *P* = .3, respectively) (Figure 1). In analogy, the percentage of CHC subjects showing a decline of HbA1c below 7.2% (the median value of the overall study population, and—by assumption—used from now as the cut‐off of glycaemic control evaluation over time) increased from 58.6% at baseline to 80.0% (*P* < .001), 78.60% (*P* = .01) and 79.4% (*P* < .001) at 48w, 96w and 120w, respectively, whereas the percentage of cirrhotics with a decline of HbA1c below 7.2% varied from 56% at baseline to 71% (*P* = .02), 57% (*P* = .8) and 54% (*P* = .4) at 48w, 96w and 120w, respectively (Figure 2). The significant and persistent amelioration of glucose metabolism in CHC versus cirrhotic patients was confirmed when considering the number of patients with HbA1c value <6.5% at the end of follow up [37/70 (52.9%) CHC vs 36/116 (31%) cirrhotic individuals (*P* = .003)]. When evaluating drugs in the two groups, no relevant changes between baseline and 120th week evaluations were observed in both CHC and cirrhosis subgroups, with the exception of 4 CHC patients (three on acarbose and one on metformin) who interrupted all anti‐diabetic treatments after DAA cure (Table S1).

**TABLE 1:** Demographic, clinical, biochemical and virological data of direct‐acting antiviral (DAA)‐treated HCV patients with type 2 diabetes

	Patients	CHC group	Cirrhosis group	*p* value
n (%)	182	70	112	
Age, years (range)	68 (41‐91)	69 (45‐90)	68 (41‐91)	0.76
Male, n (%)	98 (53.8)	34 (48.6)	64 (57.1)	0.18
BMI kg/m^2^, mean (±SD)	26.2 (4.6)	25.8 (4.6)	26.6(4.8)	0.96
AST (U/L)	67 (13‐269)	58 (13‐269)	74 (18‐245)	0.41
ALT (U/L)	80 (9‐332)	72 (9‐332)	87 (11‐212)	0.28
GGT (U/L)	91 (11‐403)	81 (11‐403)	110 (14‐386)	0.09
Bilirubin (mg/dL)	1 (0.4‐1.8)	0.9 (0.4‐1.7)	0.9 (0.5‐1.8)	0.5
Albumin (g/dL)	3.9 (2.7‐4.9)	3.9 (2.5‐4.9)	3.8 (2.7‐4.3)	**0.01**
Gamma globulin (g/dL)	1.6 (0.6‐3.2)	1.2 (0.6‐1.4)	1.7 (0.9‐3.2)	**0.002**
Liver stiffness (kPa)^a^	19.6 (10‐75)	11.1 (10‐12)	23.7 (12.8‐75)	**<0.001**
HCV genotypes				
1a	21	6 (8.5)	15 (13.4)	0.36
1b	113	42 (60)	65 (58)	0.79
2	37	18 (25.7)	21 (18.8)	0.26
3	7	2 (2.8)	5 (4.5)	0.61
4	8	2 (2.8)	6 (5.2)	0.45
Diabetes family history, n (%)	41	13 (21.4)	28 (25)	0.31
Diabetes known duration (years)	9 (1‐34)	9 (3‐24)	10 (1‐30)	0.42
FBG (mg/dL)	130 (74‐286)	134 (74‐229)	130 (80‐286)	0.23
HbA1c (g/dL)	7.2 (5‐11.2)	7.1 (5.2‐10.8)	7.2(5‐11.2)	0.76
Cholesterol (mg/dL)	159 (95‐232)	156 (95‐232)	165 (116‐222)	0.41
Triglycerides (mg/dL)	110 (58‐222)	105 (58‐222)	98 (59‐209)	0.56
Creatinine (mg/dL)	0.85 (0.4‐1.9)	0.86 (0.4‐1.9)	0.82(0.5‐1.9)	0.13
Diabetes macrovascular complications	13 (7)	3 (4.3)	10 (8.6)	0.26
Diabetes microvascular complications	42 (22.6)	13 (18.6)	29 (25)	0.31
Arterial hypertension, n (%)	107(58.8)	45 (64.3)	62 (53.5)	0.23
More than one oral hypoglycaemic drugs users, n (%)	17 (9.3)	5 (7.1)	12 (10.7)	0.42
Insulin treatment, n (%)	77 (42.3)	26 (37.1)	51 (45.5)	0.26
Teetotaler, n (%)	95 (52.2)	38	57	0.71
Mild drinkers (up to 2 AU), n (%)	63 (34.6)	29	34	
Heavy drinkers (more than 2 AU), n (%)	24 (13.2)	11	13	

Note

Bold characters identify statistically significant variables.

Abbreviations: ALT, alanine aminotransferase; AST, aspartate aminotransferase; BMI, body mass index; FBG, fasting blood glucose; GGT, gamma glutamyl transpeptidase; HCV, hepatitis C virus.

^a^ Elastographic examination was not performed in five CHC and in four cirrhotic patients due to BMI > 33kg/m^2^. All numerical parameters are expressed as median and range except for those otherwise indicated.

During the 120 weeks of post‐DAA treatment follow up, all the CHC patients had a clear and stable amelioration of the liver function parameters, and only 6/70 cases showed persistence of minimal alteration of AST and/or GGT values over time likely seen as a consequence of the diabetes‐related non‐alcoholic fatty liver disease (Table 2). Of note, no relevant fluctuation of BMI values was observed at the different time points of the follow up compared with baseline, neither in CHC or cirrhotic patients [26.3 ± 4.6 standard deviation (SD) and 26.9 ± 4.1 SD at baseline and at 120w, respectively (*P* = .7) in CHC; 25.8 ± 4.8 SD and 26.6 ± 3.9 SD at baseline and 120w, respectively (*P* = .57) in cirrhotics. No relevant change in T2DM treatments was noted during the follow up in both CHC and cirrhotic subjects. Furthermore, although the percentage of patients under insulin therapy was higher in cirrhosis compared with CHC, this difference did not reach a significant difference both at baseline and at 120w (*P* = .26 and 0.17, respectively) (Table 2).

**TABLE 2:** Liver biochemistry and liver stiffness in CHC and cirrhotic subjects at baseline and at the end of follow up (120 weeks after stopping DAA treatment)

	CHC		*P* value	Cirrhosis		*P* value
	Baseline	End of the study		Baseline	End of the study	
n	70	70		112	91^a^	
AST (U/L)	**58 (13‐269)**	**24 (11‐61)**	.**001**	**74 (18‐245)**	**32 (11‐31)**	.**001**
ALT (U/L)	**72 (9‐332)**	**16 (9‐41)**	.**001**	**87 (11‐212)**	**8 (8‐44)**	.**001**
GGT (U/L)	**81 (11‐403)**	**54 (11‐89)**	.**001**	**110 (14‐386)**	**64 (18‐91)**	.**001**
Bilirubin total (mg/dL)	0.9 (0.4‐1.7)	0.8 (0.4‐1.7)	.67	0.9 (0.5‐1.8)	0.8 (0.5‐1.8)	.45
Albumin (g/dL)	3.9 (3.5‐4.9)	4.05(3.5‐4.7)	.1	3.8 (2.7‐4.3)	3.8 (2.7‐4.1)	.1
Gamma globulin (gr/dL)	1.2 (0.6‐1.4)	1.1 (0.6‐1.3)	.9	**1.7 (0.9‐3.2)**	**1.4 (0.8‐2.2)**	.**001**
Liver stiffness (kPa)	**11.1 (10‐12)**	**7.6 (4.8‐11.8)**	**<.001**	**23.7 (12.8‐75)**	**15.6 (9.7‐69)**	.**001**
BMI kg/m^2^ mean (±SD)	26.3 (4.6)	26.9 (4.1)	.7	25.8(4.8)	26.6 (3.9)	.57
FBG (mg/dL)	134 (74‐229)	121(88‐208)	.**05**	130 (80‐286)	132(76‐288)	.7
HbA1c (g/dL)	**7.1 (5.2‐10.8)**	**6.5 (4.9‐9.3)**	**<.001**	7.2 (5‐11.2)	7.05 (5.1‐11.1)	.34
HbA1c < 6.5%, n (%)	22 (31.4)	37 (52.9)	.**01**	41 (35.3)	36 (31)	.48
Creatinine (mg/dL)	0.86 (0.4‐1.9)	0.78 (0.4‐1.8)	.31	0.82 (0.5‐1.9)	0.71 (0.6‐1.7)	.21
Arterial hypertension, n (%)	45 (64.3)	45 (64.3)	1	62 (53.5)	62 (53.5)	1
Insulin treatment, n (%)	26 (37.1)	24 (34.3)	.71	51 (45.5)	50 (44.6)	.89

Note

All numerical parameters are expressed as median and range except for those otherwise indicated. Bold characters identify statistically significant results at multivariate analysis.

Abbreviations: ALT, alanine aminotransferase; AST, aspartate aminotransferase; BMI, body mass index; FBG, fasting blood glucose; GGT, gamma glutamyl transpeptidase; HbA1c, glycated haemoglobin; PTL, platelet.

^a^ The evaluation excluded 21 patients who developed liver decompensation and or hepatocellular carcinoma during 120 weeks of follow up.

Liver stiffness values significantly decreased in all the CHC cases (*P* < .001, baseline compared with 120w time points) as well as in 91/112 cirrhotic patients (*P* = .001) (Table 2). Despite the overall improvement of both liver stiffness values and biochemical parameters (AST, ALT, GGT and gamma globulin, *P* < .001) 21/112 (18.7%) cirrhotics showed patterns of liver disease decompensation and/or HCC development during the 120 weeks of follow up. Of importance, even excluding these 21 subjects from the analysis, the glycaemic control profile did not change in both the overall study population and the cirrhosis‐subgroup at the different time points evaluated (*P* < .001, *P* = .71 and *P* = .41, respectively).

Taking into account, collectively, all the variables examined at baseline, the grade of liver damage was the only predictor of 96w and 120w significant metabolic control improvement (HbA1c < 7.2%), at multivariate logistic regression analysis (Table 3), and those patients with chronic hepatitis have a 2.5 (CI 1.066‐5.945) times greater chance of achieving glycaemic control than those patients with liver cirrhosis (*P* = .035) (Table 3). On the contrary, age, sex, BMI, GOT, GPT, GGT, albumin, gamma globulins values, alcohol intake, HCV genotype, presence of oesophageal varices, liver decompensation, diabetes duration, diabetes family history, diabetes complications and diabetic treatments (insulin vs. oral hypoglycaemic agents) did not independently predict metabolic control in this population.

**TABLE 3:** Logistic regression analyses of different variables according to glucose control (HbA1c < 7.2) at 120 weeks after the end of DAA treatment in 182 HCV/diabetic patients

Variables	Univariate model			Multivariate model		
	OR	95% CI	*P* value	OR	95% CI	*P* value
Sex	1.4	0.98‐1.05	.27	1.75	0.98‐3.52	.14
Age	1.1	0.98‐1.05	.23	1.00	0.97‐1.09	.33
BMI	0.97	0.95‐1.00	.08	1.00	0.93‐1.07	.32
Chronic hepatitis vs. cirrhosis	**2.29**	**1.17‐4.49**	.**01**	**2.51**	**1.06‐5.94**	.**03**
HCV genotypes	0.9	0.44‐1.84	.78	1.42	0.23‐8.71	.7
Presence of varices	1.34	0.70‐2.75	.34	0.98	0.41‐2.27	.96
Arterial hypertension	0.93	0.47‐1.08	.84	0.98	0.96‐1.02	.76
AST	0.99	0.97‐1.01	.53	0.96	0.82‐0.98	.66
ALT	1.00	0.99‐1.01	.70	0.94	0.76‐1.22	.26
GGT	1.00	0.99‐1.01	.56	0.91	0.74‐1.03	.15
Bilirubin	0.94	0.59‐1.48	.81	0.98	0.91‐1.21	.65
Albumin	1.66	0.79‐3.49	.18	0.68	0.24‐1.18	.37
Gamma globulin	0.92	0.61‐1.52	.77	1.27	0.60‐1.93	.47
Total cholesterol	0.99	0.98‐1.02	.63	0.98	0.97‐1.01	.88
Triglycerides	1.0	0.99‐1.23	.65	0.34	0.97‐1.03	.71
Creatinine	1.64	0.46‐3.55	.45	0.97	0.34‐2.78	.96
PTL	0.99	0.99‐1.03	.07	0.97	0.95‐1.01	.08
HCC	2.45	0.67‐7.81	.18	2.4	0.75.6.43	.27
Liver decompensation	1.09	0.61‐1.98	.77	0.98	0.45‐2.11	.76
Diabetes duration	1.3	0.72‐2.74	.47	0.9	0.86‐1.21	.5
Diabetes family history	1.04	0.96‐1.99	.06	1.56	0.71‐5.21	.11
Diabetes complications	1.61	0.51‐5.02	.40	2.1	0.78‐3.15	.72
Insulin treatment	0.74	0.53‐1.15	.08	1.7	0.82‐3.71	.14

Note

Bold characters identify statistically significant results at multivariate analysis.

Abbreviations: ALT, alanine aminotransferase; AST, aspartate aminotransferase; BMI, body mass index; CI, confidence interval; GGT, gamma glutamyl transpeptidase; HCC, hepatocellular carcinoma; OR, odds ratio; PTL, platelet.

## 5 DISCUSSION

The relationship between HCV chronic infection and T2DM is an important and largely debated argument, which involves specialists of different areas because of its relevant clinical implications and challenging physio‐pathological aspects.^25^, ^26^, ^27^, ^28^ After the recent advent of DAA‐based therapy which is able to quickly and definitively cure the HCV infection, the interest about interplay between HCV and diabetes is even greater. A major question arising is whether HCV elimination might have any effect on metabolic control in T2DM patients. However, contrasting data are available in this field, because the studies performed so far show substantial differences in the enrolled patient populations and limited durations of the post‐DAA‐treatment follow up.^29^, ^30^ Our study showed that subjects with chronic hepatitis had a significant and persistent amelioration of the glycaemic control and antidiabetic therapy could even be stopped in four patients. On the contrary, patients with compensated cirrhosis showed only a transient improvement, with a HbA1c rebound to pretreatment values 1 year after stopping DAA therapies. Thus, virus elimination has not only an extraordinarily positive impact on the liver disease in these individuals but also a long‐term beneficial effect on the diabetes course.

Our findings have several, important implications. Considering that glucose control usually tends to deteriorate over time,^31^ the significant and stable decrease of HbA1c levels—without implementing hypoglycaemic treatment for over 2 years—in HCV‐cured CHC subjects has an evident clinical relevance and appears to indirectly confirm the pathogenic role of that virus in worsening glucose metabolism in subjects with diabetes or even in favouring or accelerating diabetes development.

Our results show a persistent amelioration of glucose control together with normalization of liver biochemistry and no evidence of liver disease progression in most SVR patients, thus suggesting an overall favourable outcome of the liver disease in these individuals possibly due to both virus elimination and improvement of metabolic disorders. The entire scenario appears to be different if DAA therapy is started when cirrhosis is already established in HCV/diabetic patients. In fact, the virus elimination occurring in patients with compensated cirrhosis appeared to be associated with an improvement of glucose control in the first year after DAA therapy, but subsequently, this association disappeared, and the HbA1c values returned to be comparable with the pretreatment levels.

Why HCV clearance has a different impact on the glucose metabolism of patients with chronic hepatitis compared with patients with cirrhosis is an interesting—but difficult to answer—question.

The liver is not the only HCV target, and a quite large number of extra‐hepatic manifestations are frequently observed during the course of this viral infection. Considering the potent effects of DAAs, which suddenly switch off a chronic inflammatory status persisting for years or even decades prior to the therapy, one might argue that the reduction of the chronic inflammation levels may have had a positive impact on diabetes control in the short run, independently of the degree of liver damage.^32^, ^33^, ^34^ Of note, a transient benefit of HCV extrahepatic manifestation was very recently reported in HCV patients with cutaneous psoriasis who had a dramatic amelioration of cutaneous lesions under DAA treatment, but almost all the cases showed a similarly dramatic worsening of their skin disease, which—24 weeks after stopping treatment—returned comparable with the basal time.^10^

To explain why the HCV recovery has only an initial, transient benefit on glucose metabolism in cases with advanced liver disease, one must consider both the multifactorial pathophysiological basis of diabetes and the large architectural, vascular and functional rearrangements occurring in the liver once cirrhosis is developed. Insulin resistance, muscle sarcopenia, deficit in hormones clearance and a ‘toxic’ effect on pancreatic islets are all suggested mechanisms determining an impairment of glucose metabolism in patients with liver cirrhosis, thus potentially contributing to the lack of long‐term metabolic benefits of DAA treatment observed in these subjects.^33^, ^34^, ^35^

Several potential limitations should be taken into account when interpreting our results, including the lack of measurements of insulin, C‐peptide, and HOMA‐IR as well as of inflammatory markers, which could have added useful information to explain our observations.

The recruitment of all consecutively treated T2DM individuals from a cohort of DAA treated subjects and the length of follow up are among the strengths of this study, rendering of particular relevance the emerged results. We are now continuing to follow the study population, and we are planning to report further data after at least 5 years of follow up, to continue providing information about the impact of DAA‐based cure on both diabetes and liver disease of HCV/diabetic subjects.

## CONFLICT OF INTEREST

The authors declare they have no conflict of interest.

## Supporting information

Table S1
