# Supplementary material for: Over time evaluation of glycaemic control in direct‐acting antiviral‐treated hepatitis C virus/diabetic individuals with chronic hepatitis or with cirrhosis
Source: Liver Int. 2021 May 26;41(9):2059–67. doi: 10.1111/liv.14905 (PMC8506140; doi:10.1111/liv.14905)
Supplement: Supplementary file 1 — Table S1 [file LIV-41-2059-s001.docx]

**Supplementary table 1: Anti-diabetic treatments in CHC and cirrhotic subjects at baseline and at the end of follow up (120 weeks after stopping DAA treatment)**

|  | **Chronic Hepatitis** | | **Cirrhosis** | |
| --- | --- | --- | --- | --- |
|  | *Baseline* | *End of the study* | *Baseline* | *End of the study* |
| Diet only n (%) | 0 | 4 (5.7) | 0 | 0 |
| Metformin n (%) | 24 (34.3) | 22 (31.4) | 33 (28.4) | 34 (29.3) |
| SU/repaglinide n (%) | 11 (15.7) | 11 (15.7) | 13 (11.2) | 15 (12.9) |
| DPP4i n (%) | 1 (1.4) | 2 (2.8) | 3 (2.6) | 4 (3.4) |
| SGLT2i n (%) | 0 | 1 (1.4) | 0 | 0 |
| Acarbose n (%) | 7 (10) | 5 (7.1) | 14 (12.1) | 13 (11.2) |
| GLP1RAs n (%) | 1 (1.4) | 1 (1.4) | 0 | 0 |
| Insulin therapy n (%) | 16 (37.1) | 24 (34.3) | 51 (45.5) | 50 (44.6) |

SU (Sulfonylureas), DPP4i (Dipeptidyl-peptidase inhibitor), GLP1RAs (glucagon like peptide 1 receptor agonists), SGLT2i (sodium-glucose cotransporter-2 inhibitor)
